# Effects of variation in resource acquisition during different stages of the life cycle on life‐history traits and trade‐offs in a burying beetle

**DOI:** 10.1111/jeb.13388

**Published:** 2018-10-27

**Authors:** Jon Richardson, Per T. Smiseth

**Affiliations:** ^1^ Institute of Evolutionary Biology University of Edinburgh Edinburgh UK

**Keywords:** life history, *Nicrophorus vespilloides*, resource acquisition, resource allocation, trade‐offs

## Abstract

Individual variation in resource acquisition should have consequences for life‐history traits and trade‐offs between them because such variation determines how many resources can be allocated to different life‐history functions, such as growth, survival and reproduction. Since resource acquisition can vary across an individual's life cycle, the consequences for life‐history traits and trade‐offs may depend on when during the life cycle resources are limited. We tested for differential and/or interactive effects of variation in resource acquisition in the burying beetle *Nicrophorus vespilloides*. We designed an experiment in which individuals acquired high or low amounts of resources across three stages of the life cycle: larval development, prior to breeding and the onset of breeding in a fully crossed design. Resource acquisition during larval development and prior to breeding affected egg size and offspring survival, respectively. Meanwhile, resource acquisition at the onset of breeding affected size and number of both eggs and offspring. In addition, there were interactive effects between resource acquisition at different stages on egg size and offspring survival. However, only when females acquired few resources at the onset of breeding was there evidence for a trade‐off between offspring size and number. Our results demonstrate that individual variation in resource acquisition during different stages of the life cycle has important consequences for life‐history traits but limited effects on trade‐offs. This suggests that in species that acquire a fixed‐sized resource at the onset of breeding, the size of this resource has larger effects on life‐history trade‐offs than resources acquired at earlier stages.

## 1 INTRODUCTION

Understanding the consequences of individual variation in resource acquisition is an important problem in life‐history evolution given that such variation can influence the expression of fundamental life‐history traits. This is because how many resources an individual acquires determines how many can be allocated to different life‐history functions. As such, individuals that acquire more resources (often termed “high‐quality individuals”) typically show increased investment in traits such as growth, survival and reproduction (e.g. Hayward, Rickard, & Lummaa, 2013; Monaghan, 2008; Nager, Ruegger, & van Noordwijk, 1997; Nagy & Holmes, 2005; Zanette, Clinchy, & Smith, 2006). Furthermore, individual variation in resource acquisition may affect trade‐offs between life‐history traits such as the trade‐offs between the number and size of offspring and between current and future reproduction. The reason for this is that these functions compete for the same pool of limited resources (Flatt & Heyland, 2011; van Noordwijk & de Jong, 1986; Roff, 2002; Smith & Fretwell, 1974), meaning that any increase in allocation towards one function should be associated with a decrease in allocation towards the other (Stearns, 1992). Individual variation in resource acquisition can affect life‐history trade‐offs by masking the negative correlations that are expected when individuals allocate limited resources between mutually exclusive functions (Lim, Senior, & Nakagawa, 2014; van Noordwijk & de Jong, 1986; Stearns, 1992).

Individuals often acquire resources during different stages of their life cycle, and the amount of resources that an individual can invest in life‐history functions can therefore vary due to variation in resource availability during different stages of the life cycle. This can have important consequences for how resource acquisition affects life‐history traits and trade‐offs between them. For instance, limitation of resources during a particular stage of the life cycle may have a greater impact on life‐history traits than limitation at other stages. Likewise, limitation of resources during different stages of the life cycle may be associated with effects on different life‐history traits. Previous work has highlighted the importance of sensitive stages of the life cycle during which there are particularly strong effects of resource limitation (e.g. Hopwood, Moore, & Royle, 2013; Kotrschal, Szidat, & Taborsky, 2014; Lindström, 1999; Metcalfe & Monaghan, 2001; Stearns & Sage, 1980; Wong & Kölliker, 2014). This may reflect that individuals cannot compensate for the effects of resource limitation during certain stages of the life cycle, leading to subsequent long‐term consequences for allocation to life‐history functions. The effects of variation in resource acquisition at one stage of the life cycle on life‐history traits may also interact with the effects of variation in resource acquisition at another stage (e.g. Barrett, Hunt, Moore, & Moore, 2009; Briga, Koetsier, Boonekamp, Jimeno, & Verhulst, 2017; Hopwood, Moore, & Royle, 2014; Taborsky, 2006; Wong & Kölliker, 2014; Zajitschek, Hunt, Jennions, Hall, & Brooks, 2009). Finally, controlling for variation in resource acquisition during sensitive stages can reveal the negative correlations between life‐history traits in a trade‐off as predicted by life‐history theory (e.g. Brown, 2003; King, Roff, & Fairbairn, 2011; Smiseth, Andrews, Mattey, & Mooney, 2014). Thus, there is now a need for more studies to examine the potential effects of individual variation in resource acquisition on life‐history traits and trade‐offs through manipulation of resource acquisition across multiple stages of the life cycle.

We examined the effects of individual variation in resource availability during different stages of the life cycle on life‐history traits and trade‐offs in the burying beetle *Nicrophorus vespilloides*. This species is a tractable system for examining the effects of variation in resource acquisition because it is straightforward to experimentally control and manipulate resource acquisition during different stages of the life cycle (Smiseth et al., 2014). *Nicrophorus vespilloides* rear their larvae on the carcasses of small vertebrates that parents prepare by removing fur, rolling into a ball and applying oral and anal secretions that prevent decay (Arce, Johnston, Smiseth, & Rozen, 2012; Scott, 1998). The carcass represents the sole source of food for developing larvae, but is acquired by the parents who search for suitable carcasses, which they secure via interspecific competition (Safryn & Scott, 2000; Scott, 1994). Thus, the size of the resource acquired determines the amount of resources that a breeding beetle has for investment in its current brood (Smiseth et al., 2014). In addition, the amount of resources acquired during larval development has consequences for adult body size given that adult body size is influenced by larval size at dispersal (Bartlett & Ashworth, 1988; Lock, Smiseth, & Moore, 2004). Furthermore, nonbreeding adults acquire resources from their environment, leading to variation in the nutritional state of individuals prior to breeding. Previous work demonstrates that variation in resource acquisition has important consequences for life‐history traits such as growth, survival and reproductive success (e.g. Bartlett & Ashworth, 1988; Gray, Richardson, Ratz, & Smiseth, 2018; Hopwood et al., 2013; Lock et al., 2004; Steiger, Richter, Müller, & Eggert, 2007). In addition, controlling for variation in resource acquisition can reveal trade‐offs between life‐history traits. For instance, the trade‐off between size and number of offspring is influenced by both carcass size (Smiseth et al., 2014) and female nutritional condition (Steiger et al., 2007). However, it is unclear whether resource limitation during different stages can have differential and/or interactive effects on life‐history traits and how important variation in resource acquisition across life stages is for the expression of life‐history trade‐offs.

In this study, we manipulated the amount of resources acquired by female *N. vespilloides* across three stages of the life cycle: during larval development, prior to breeding as an adult, and at the onset of breeding. We assigned females to either high or low amounts of resources at each stage in a fully crossed design. We examined the subsequent effects of variation in resource acquisition at these stages on a suite of life‐history traits associated with reproduction (clutch size, egg size, hatching success, brood size, brood mass, offspring mass, survival of offspring to eclosion, and offspring lifespan) and investment to self‐maintenance/future reproduction (female mass change and female lifespan). We also examined the effects of resource acquisition on the relationship between life‐history traits in putative trade‐offs. Specifically, we examined the trade‐off between the size and number of offspring and between current and future reproduction (i.e. total brood mass and female lifespan, respectively). Our first prediction was that variation in resource acquisition during different stages of the life cycle would have consequences for different life‐history traits. We also predicted that resource limitation during larval development and at the onset of breeding would have the strongest effects on life‐history traits and would affect a greater number of traits given that variation at these stages has fixed consequences (Smiseth et al., 2014; Steiger, 2013). Our second prediction was that there would be a positive relationship between traits in a putative trade‐off when we excluded information on individual variation in resource acquisition. In contrast, we predicted negative phenotypic correlations between these traits (i.e. evidence for trade‐offs) when we included information on individual variation in resource acquisition. This is because variation in resource acquisition is expected to mask variation in allocation strategies (van Noordwijk & de Jong, 1986). Finally, we predicted that the negative phenotypic correlation between traits would be stronger for females assigned to low‐resource acquisition treatments than for females assigned to high‐resource acquisition treatments given that prior studies show that trade‐offs are more pronounced when resources are limited (Smiseth et al., 2014).

## 2 MATERIALS AND METHODS

### 2.1 Beetle husbandry

We used 4th‐ and 5th‐generation laboratory‐reared beetles from lines originally collected in Edinburgh, UK. Beetles were maintained at 20°C, under a 16:8‐hr light:dark cycle. Nonbreeding beetles were housed individually in clear, plastic containers (12 × 8 × 2 cm) filled with 1 cm of moist soil and fed raw, organic beef twice weekly.

### 2.2 Experimental design

**Figure 1:**
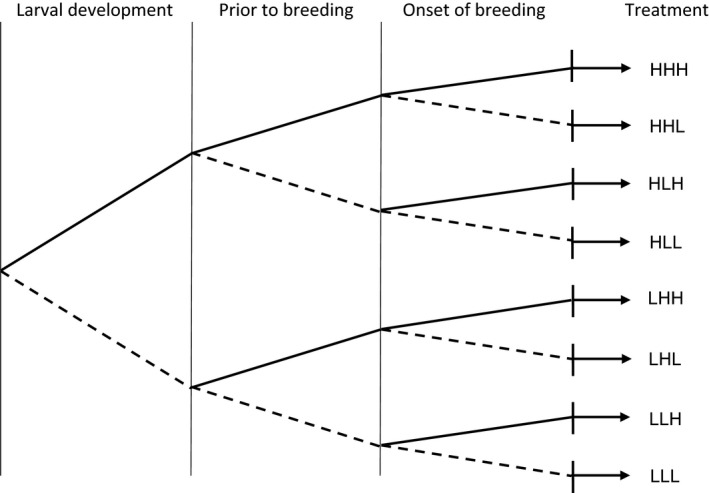
Graphical illustration of experimental design. Shown from left to right are the different stages of the life cycle and the treatment code. The level of resources an individual was able to acquire in each stage is indicated as either high (H) or low (L). Solid lines reflect nonlimited resource acquisition, and dashed lines reflect limited resource acquisition

In our study, we manipulated resource acquisition across three stages of the life cycle: during larval development, prior to breeding as adults, and at the onset of breeding (see Figure 1 for a graphical illustration of the experimental design). All experimental treatments had two levels: “low” (L) and “high” (H), reflecting differences in the amount of resources that an individual female acquired in a given stage. All individuals were exposed to one of the two treatment levels for each stage across all three stages of the life cycle. The fully crossed design resulted in eight treatment combinations (number of individuals in brackets): HHH (*n* = 27), HHL (*n* = 20), HLH (*n* = 23), HLL (*n* = 21), LHH (*n* = 28), LHL (*n* = 20), LLH (*n* = 28) and LLL (*n* = 20).

### 2.3 Resource acquisition during larval development

We manipulated resource acquisition during larval development by varying the amount of time that individual larvae were able to feed from the carcass. Larvae in the “low” resource group were removed from the carcass when they had reached a mass of 100–150 mg, and larvae in the “high” resource group were removed when they reached a mass of 200–250 mg. We did this to limit the amount of resources larvae could acquire during this stage, which has consequences for adult body size in this species (Bartlett & Ashworth, 1988; Lock et al., 2004). Removing larvae from the carcass at different times therefore generates adults that differ in size (Pilakouta, Halford, Rácz, & Smiseth, 2016a; Pilakouta, Richardson, & Smiseth, 2015, 2016b; Pilakouta & Smiseth, 2016; Steiger, 2013). Once removed from the carcass, larvae were placed in individual containers (12 × 8 × 2 cm) filled with moist soil and left to pupate and eclose as adults. At eclosion, approximately 20 days later, we measured the pronotum width of all adult females to confirm that our treatment had influenced resource acquisition during larval development. As predicted, there was a substantial difference in the mean (±SD) pronotum width of females from the two groups (*F*_1, 185_ = 1672.7, *p* < 0.001): 4.04 (±0.24) mm for the “low” resource females and 5.33 (±0.24) mm for “high” resource females. There was no overlap in the range of pronotum widths for “low” (3.50–4.59 mm) and “high” resource females (4.99–6.00 mm). These sizes are similar to those observed in laboratory populations and beetles collected in the field (Steiger, 2013).

### 2.4 Resource acquisition prior to breeding as an adult

We manipulated resource acquisition prior to breeding as an adult by restricting food availability during that period. In the “low” resource group, females were not fed for 7 days prior to breeding. By contrast, females in the “high” resource group were fed three times in the 7 days prior to breeding. We only exposed females to restriction in food availability after they had reached sexual maturity at 10 days post‐eclosion to prevent any potential effects of resource limitation on the timing of sexual maturation. The purpose of these treatments was to generate females that differed in their nutritional condition prior to breeding as measured by their prebreeding mass. As intended, females in the “low” resource group lost mass in the 7 days prior to breeding, whereas there was no change in the mass of females in the “high” resource group. As a result, there was a significant difference in the mean (±SD) mass of females in the different feeding treatment both within (*F*_1, 185_ = 21.6, *p* < 0.001) and between size classes (*F*_3, 183_ = 572.2, *p* < 0.001): 100.22 (±11.89) mg for ‘LL‐’ females; 130.79 (±12.91) mg for “LH‐” females; 216.46 (±17.20) mg for “HL‐” females; and 260.21 (±19.41) mg for “HH‐” females.

### 2.5 Resource acquisition at the onset of breeding

In order to manipulate resource acquisition during breeding, we provided females with either ‘low’ (a 3–8‐g mouse carcass) or “high” breeding resources (a 23‐ to 28‐g carcass). This simulates a situation in the wild where a female has acquired a carcass for breeding, although we note that our design excludes potential effects due to competition between females over carcasses. We chose these sizes based on previous work showing that *N. vespilloides* breeds on carcasses ranging in size from 1 to 40 g and that brood size is regulated to match carcass size when breeding on a carcass smaller than 10 g (Müller, Eggert, & Furlkröger, 1990; Smiseth & Moore, 2002). On the day of mating, we first measured the prebreeding mass of each female, which we later used to estimate the female's mass change over the breeding attempt. Mating was initiated by placing each experimental female in a transparent plastic container (11 × 11 × 3 cm) together with an unrelated virgin male for 8 hr (Botterill‐James, Ford, While, & Smiseth, 2017; Ford, Henderson, & Smiseth, 2018; Gray et al., 2018). This design was used to ensure that females received sufficient sperm for fertilization and so that they could breed on their own without male assistance. We excluded males during the actual breeding attempt to remove any confounding effects caused by the male's consumption of the carcass or assistance in parental care. After mating, we transferred experimental females to a larger transparent plastic container (17 × 12 × 6 cm) lined with 1 cm of moist soil for breeding. To initiate breeding, we provided females with a freshly thawed mouse carcass of the appropriate size depending on the treatment to which they had been assigned (see above).

From the day of mating and onwards, we checked for eggs twice daily. Immediately before the eggs were expected to hatch (which takes about 59 hr at 20°C; Smiseth, Ward, & Moore, 2006), we scanned the bottom of each container using a CanoScan 9000F Mark II scanner (Canon, Tokyo). We did this to record the number and size of eggs (Ford & Smiseth, 2016). For each scanned image, we counted the number of visible eggs as a measure of clutch size. Because each container has only a very thin layer of soil, the number of eggs visible at the bottom of the container is strongly correlated with the actual clutch size (Monteith, Andrews, & Smiseth, 2012). We also measured the length and width of up to six randomly selected eggs in pixels using ImageJ (Abràmoff, Magalhães, & Ram, 2004; Monteith et al., 2012). We then converted these measurements to metric length (mm) and calculated a prolate spheroid volume (*V)* for each egg using the equation *V *= (1/6) π*w* ^2^ *l*, where *w* is the width and *l* is the length of the egg (Berrigan, 1991). We used these measures of clutch size and egg size for each brood to examine the trade‐off between the number and size of eggs. We left females to rear their brood undisturbed until the larvae dispersed from the carcass approximately 7 days later.

When all larvae had dispersed from the carcass, we weighed each female again to measure her post‐breeding mass. We then calculated the mass change over the breeding attempt for each female by subtracting her prebreeding mass (see above) from her post‐breeding mass. Females were then transferred to individual containers (12 × 8 × 2 cm) filled with 1 cm of moist soil and maintained following the protocol for beetles in the stock population (see above). Females were checked twice weekly until death to record their lifespan. At the dispersal stage, we also recorded the number of unhatched eggs visible at the bottom of the box, the number of dispersing larvae and the total mass of the brood. We estimated hatching success by first subtracting the number of unhatched eggs from the clutch size (see above) and then dividing this estimate of the number of hatched eggs by the clutch size. We also calculated average larval mass in each brood by dividing the total brood mass by the number of larvae in the brood. We used our measures of the number of larvae and the average mass of larvae in each brood to examine the trade‐off between the number and size of offspring at larval dispersal. Similarly, we used our measures of total brood mass and lifespan for each female to examine the trade‐off between current and future reproduction. We then placed the larvae from each brood into transparent plastic containers (17 × 12 × 6 cm) filled with moist soil. Approximately 20 days later, we recorded the number of individuals that successfully eclosed. At this stage, up to six beetles from each brood were placed into individual containers (12 × 8 × 6 cm) and checked twice a week until death to record average lifespan of offspring.

### 2.6 Statistical analyses

All analyses were performed using R v.3.5.1 (R Core Team 2018). To examine the effects of variation in resource acquisition across different life stages on life‐history traits and the trade‐offs between them, we performed three sets of analyses. In the first set of analyses, we used a univariate linear model approach to test the effects of variation in resource acquisition at different stages of the life cycle on the expression of life‐history traits. The purpose of these analyses was to determine whether variation in individual resource acquisition during different stages of the life cycle had differential and/or interactive effects on life‐history traits. In the second set of analyses, we excluded information on individual variation in resource acquisition and examined the relationship between (a) size and number of offspring both at the egg‐laying stage and at larval dispersal, and (b) current and future reproduction based on measures of total brood mass and female lifespan, respectively. The purpose of this analysis was to determine whether there was a positive or negative relationship between life‐history traits in a putative trade‐off when information on variation in resource acquisition was not included. In our final set of analyses, we examined the same trade‐offs while including information on individual variation in resource acquisition at different stages of the life cycle using a bivariate linear mixed model approach. The purpose of this analysis was to test whether the relationship between life‐history traits in a putative trade‐off changed when explicitly controlling for variation in resource acquisition between individuals, as expected if individual variation in resource acquisition masks life‐history trade‐offs (van Noordwijk & de Jong, 1986).

For the univariate analyses of life‐history traits, we used general linear models for continuous traits with normally distributed errors (egg size, brood size, brood mass, average offspring mass, female mass change, female lifespan and offspring lifespan) and generalized linear models for count data with Poisson errors (clutch size) and proportional data with binomial errors (hatching success and eclosion success). Univariate models included the following factors: resource acquisition treatment during larval development (H or L), resource acquisition treatment prior to breeding as an adult (H or L), and resource acquisition treatment at the onset of breeding (H or L), as well all corresponding two‐way interactions. The three‐way interaction between treatments was not significant for any traits and was therefore removed from the analyses. To account for multiple testing, we used false discovery rate corrections (Benjamini & Hochberg, 1995). For bivariate analyses of life‐history trade‐offs in which information on resource acquisition was excluded, we included both traits in a putative trade‐off as dependent variables and the identity of the female as a random effect. For bivariate models that included information on individual variation in resource acquisition, we also included the same factors and interaction effects as those described for the univariate models (see above).

## 3 RESULTS

### 3.1 Effects of resource acquisition on life‐history traits

Resource limitation during larval development had a significant effect on egg size as females that acquired fewer resources during larval development laid smaller eggs than females that acquired more resources during larval development (Table 1). However, individual variation in resource acquisition during larval development had no effect on any other traits (Table 1). Variation in resource acquisition prior to breeding as an adult (i.e. female nutritional state) had a significant effect on the amount of mass that females gained during breeding with starved females gaining more mass than nonstarved females (Table 1). In addition, there was a significant effect on the proportion of offspring in the brood surviving to eclosion with starved females having fewer offspring alive at eclosion when breeding on large carcasses (see below; Table 1). There were no effects of resource acquisition during this stage on other traits (Table 1). Resource acquisition at the onset of breeding (i.e. carcass size) had significant effects on the size and number of offspring. Females breeding on large carcasses laid significantly larger clutches and larger eggs than females breeding on small carcasses (Table 1). In addition, females breeding on large carcasses produced broods with more offspring that were heavier in terms of both the total brood mass and the mean mass of the larvae than females breeding on small carcasses (Table 1). There were no effects of resource acquisition at the onset of breeding on other traits (Table 1).

**Table 1:** Effects of variation in resource acquisition during larval development (which influenced adult body size), prior to breeding as an adult (nutritional state), and at the onset of breeding (carcass size) and their two‐way interactions on life‐history traits in *Nicrophorus vespilloides*. We provide parameter estimates (±SE), test statistics (LR χ^2^) and *p*‐values from univariate linear models. We present raw *p*‐values with bold type indicating *p*‐values that remained significant after false discovery rate correction

Trait	Estimate (±SE)	LR χ^2^	*p*‐value
Larval development (adult body size)			
Clutch size	−0.09 (0.14)	0.48	0.48
Egg size (mm^3^)	−0.31 (0.08)	25.1	**<0.001**
Hatching success (%)	−0.13 (0.50)	0.064	0.80
Brood size	−3.24 (1.88)	2.68	0.10
Brood mass (g)	−1.21 (0.32)	2.95	0.085
Offspring mass (g)	−0.02 (0.01)	1.00	0.31
Female mass change (g)	−0.003 (0.01)	0.40	0.53
Eclosion success (%)	−0.02 (0.43)	0.26	0.60
Female lifespan (days)	3.31 (3.00)	1.21	0.27
Offspring lifespan (days)	4.76 (2.30)	4.04	0.044
Prior to breeding (nutritional state)			
Clutch size	−0.22 (0.12)	2.64	0.10
Egg size (mm^3^)	−0.11 (0.08)	3.30	0.17
Hatching success (%)	0.43 (0.50)	0.73	0.39
Brood size	−3.02 (2.05)	3.35	0.066
Brood mass (g)	−0.84 (0.35)	4.87	0.027
Offspring mass (g)	−0.003 (0.01)	3.02	0.081
Female mass change (g)	0.05 (0.01)	91.7	**<0.001**
Eclosion success (%)	−3.12 (0.38)	64.2	**<0.001**
Female lifespan (days)	2.83 (3.10)	0.82	0.36
Offspring lifespan (days)	−4.38 (2.40)	3.15	0.075
Onset of breeding (carcass size)			
Clutch size	−0.33 (0.14)	5.13	**0.024**
Egg size (mm^3^)	−0.24 (0.08)	10.8	**<0.001**
Hatching success (%)	−0.34 (0.54)	0.38	0.53
Brood size	−4.29 (1.98)	7.16	**0.0074**
Brood mass (g)	−2.18 (0.34)	27.6	**<0.001**
Offspring mass (g)	−0.08 (0.01)	49.7	**<0.001**
Female mass change (g)	−0.01 (0.01)	1.75	0.18
Eclosion success (%)	0.62 (0.48)	0.21	0.64
Female lifespan (days)	4.57 (3.20)	2.02	0.15
Offspring lifespan (days)	2.57 (2.50)	1.03	0.31
Larval development × prior to breeding			
Clutch size	−0.04 (0.16)	0.015	0.90
Egg size (mm^3^)	0.43 (0.09)	26.2	**<0.001**
Hatching success (%)	−0.03 (0.61)	0.002	0.96
Brood size	−0.79 (2.25)	0.004	0.94
Brood mass (g)	0.52 (0.39)	0.8	0.36
Offspring mass (g)	0.02 (0.01)	1.26	0.26
Female mass change (g)	−0.03 (0.01)	18.6	**<0.001**
Eclosion success (%)	0.85 (0.48)	3.08	0.079
Female lifespan (days)	−7.30 (3.70)	3.93	0.047
Offspring lifespan (days)	−2.91 (2.90)	1.01	0.32
Larval development × onset of breeding			
Clutch size	−0.02 (0.18)	0.019	0.88
Egg size (mm^3^)	−0.06 (0.09)	0.12	0.72
Hatching success (%)	−0.53 (0.59)	0.80	0.37
Brood size	2.06 (2.25)	1.60	0.35
Brood mass (g)	0.70 (0.39)	1.30	0.25
Offspring mass (g)	0.01 (0.01)	0.93	0.33
Female mass change (g)	−0.01 (0.01)	0.92	0.34
Eclosion success (%)	−1.39 (0.51)	1.55	0.21
Female lifespan (days)	−5.25 (3.70)	1.98	0.15
Offspring lifespan (days)	−6.57 (2.90)	5.04	0.0247
Prior to breeding × onset of breeding			
Clutch size	0.10 (0.17)	0.24	0.62
Egg size (mm^3^)	−0.09 (0.09)	2.21	0.31
Hatching success (%)	0.43 (0.61)	0.48	0.48
Brood size	1.33 (2.24)	0.75	0.21
Brood mass (g)	0.72 (0.39)	2.38	0.12
Offspring mass (g)	0.03 (0.01)	0.99	0.31
Female mass change (g)	0.01 (0.01)	3.87	0.049
Eclosion success (%)	2.71 (0.51)	33.1	**<0.0001**
Female lifespan (days)	0.82 (3.70)	0.04	0.82
Offspring lifespan (days)	5.64 (2.90)	3.71	0.053

In addition to the main effects of resource acquisition on life‐history traits, we also found that the effects of resource acquisition at one stage interacted with those at other stages. For instance, there was a significant effect of the interaction between resources acquired during larval development and resources acquired prior to breeding as an adult on egg size (Table 1). This interaction effect indicated that those females that acquired fewer resources during larval development and that were also starved prior to breeding produced larger eggs than those females that acquired fewer resources during larval development but were not starved prior to breeding. In addition, there was a significant effect of the interaction between resources acquired prior to breeding and resources acquired at the onset of breeding on the number of offspring in a brood that survived to eclosion. This effect occurred because starved females breeding on large carcasses had fewer offspring surviving to eclosion than starved females breeding on small carcasses (Table 1).

### 3.2 Effects of resource acquisition on life‐history trade‐offs

**Figure 2:**
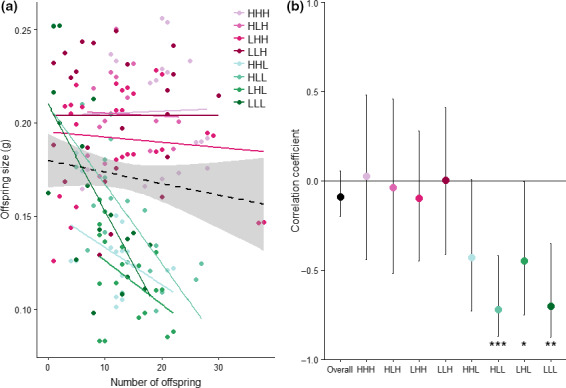
Effects of variation in resource acquisition on the trade‐off between number and size of offspring at larval dispersal. Females acquired either high (H) or low (L) resources during larval development, prior to breeding as an adult, and at the onset of breeding in a fully crossed design to give eight treatments (HHH, HHL, HLH, HLL, LHH, LHL, LLH and LLL). Colours represent the specific treatment that a female experienced. (a) The coloured circles represent the number and mean size of offspring produced by different females. The black dashed line represents the relationship between number and size of offspring when information on individual variation in resource acquisition is excluded (±95% CI). The coloured lines represent this relationship for each treatment when information on resource acquisition is included. Pink colours represent females breeding on large carcasses, and green colours represent females breeding on small carcasses. (b) Correlation coefficients between number and size of offspring at larval dispersal (±95% CI). The black circle represents the correlation coefficient for the whole data set when information on individual variation in resource acquisition is excluded, and the coloured circles represent the correlation coefficient for each treatment. Treatments for which the correlation coefficient differs significantly from zero are indicated with asterisks (*p < 0.05, **p < 0.01, ***p < 0.001)

There was no relationship between the number and size of offspring at the time of larval dispersal when we excluded information on individual variation in resource acquisition (LR χ^2^ = 1.61, *p* = 0.20). However, when we included information on individual variation in resource acquisition, there was a negative relationship between the number of larvae and mean larval mass at the time of dispersal, indicative of a trade‐off between the number and size of offspring (Table 2; Figure 2). This trade‐off was affected by the amount of resources that females acquired at the onset of breeding (i.e. carcass size), as there was a significant negative relationship between the size and number of offspring at larval dispersal when females bred on a small carcass but not when females bred on a large carcass (Table 2; Figure 2). Thus, females breeding on small carcasses produced smaller offspring as brood size increased, whereas this was not the case for females breeding on large carcasses. The trade‐off between the size and number of offspring at larval dispersal was not affected by the amount of resources a female acquired during larval development or the resources acquired prior to breeding as an adult (Table 2). Similarly, there was no effect of interactions between resources at each stage on the trade‐off between the size and number of offspring (Table 2).

**Table 2:** Effects of variation in resource acquisition during larval development (i.e. adult body size), prior to breeding as an adult (i.e. nutritional state), and at the onset of breeding (i.e. carcass size) and their two‐way interactions on life‐history trade‐offs in *Nicrophorus vespilloides*. We provide test statistics (LR χ^2^) and *p*‐values from bivariate linear mixed models examining the trade‐off between offspring size and number at larval dispersal, between egg size and number and between brood mass and lifespan (as proxies for current and future reproduction, respectively). Statistically significant *p*‐values are indicated in bold type

	Offspring size vs. number at larval dispersal		Egg size vs. number		Brood mass vs. lifespan	
	LR χ^2^	*p*‐value	LR χ^2^	*p*‐value	LR χ^2^	*p*‐value
Main effects						
Larval development (adult size)	2.9	0.08	0.3	0.57	1.7	0.18
Prior to breeding (nutritional state)	2.2	0.13	0.1	0.81	2.3	0.12
Onset of breeding (carcass size)	4.8	**0.027**	3.7	0.051	0.7	0.39
Interactions						
Larval development × prior to breeding	0.1	0.72	0.5	0.46	3.6	0.057
Larval development × onset of breeding	0.8	0.35	0.6	0.41	0.7	0.37
Prior to breeding × onset of breeding	0.3	0.54	0.2	0.67	0.1	0.73

There was no evidence for a trade‐off between number and size of eggs. There was no relationship between clutch size and egg size when information on resource acquisition was excluded (LR χ^2^ = 0.47, *p* = 0.49). Likewise, there was no relationship between clutch size and egg size when information on individual variation in resource acquisition was included, and this was the case regardless of whether we focused on resource acquisition during larval development, prior to breeding as an adult or at the onset of breeding (Table 2). There was also no effect of the interactions between stages on the relationship between clutch size and egg size (Table 2).

There was no evidence for a relationship between brood mass and female lifespan (i.e. current and future reproduction, respectively), when we excluded information on individual variation in resource acquisition (LR χ^2^ = 0.69, *p* = 0.40). Likewise, including information on resource acquisition during larval development, prior to breeding or during breeding had no effect on the relationship between brood mass and lifespan (Table 2). There was no evidence for interactions between stages on brood mass and female lifespan (Table 2).

## 4 DISCUSSION

In this study, we investigated effects of individual variation in resource acquisition during different stages of the life cycle on life‐history traits and trade‐offs between them in females of the burying beetle *N. vespilloides*. We found that resource acquisition during larval development (which influenced female body size), prior to breeding as an adult (i.e. female nutritional state), and at the onset of breeding (i.e. carcass size) affected different life‐history traits (see details below). We found no evidence for life‐history trade‐offs when we excluded information on individual variation in resource acquisition. However, there was a trade‐off between number and size of offspring when we included information on resource acquisition. In contrast, there was no evidence for a trade‐off between number and size of eggs or between brood mass and lifespan (our proxy measures for current and future reproduction, respectively) regardless of whether we excluded or included information on individual variation in resource acquisition. Below we provide a more detailed discussion of our results.

As expected, variation in resource acquisition during different stages of the life cycle affected different life‐history traits. Resource acquisition during larval development influenced egg size with females acquiring fewer resources during larval development producing smaller eggs. This finding likely reflects that individuals that acquired fewer resources as larvae develop into smaller adults (Bartlett & Ashworth, 1988; Lock et al., 2004) and smaller females may lay smaller eggs due to morphological or physiological constraints, such as the amount of available body space for the egg, the size of the ovipositor or the rate of resource transfer from mother to egg (Sakai & Harada, 2001; Steiger, 2013; Yanagi & Tuda, 2012). Meanwhile, resource acquisition prior to breeding influenced mass gain over the reproductive attempt with starved females gaining more mass than nonstarved females. This result likely reflects that resource acquisition prior to breeding determines female nutritional condition and starved females may feed more from the carcass than nonstarved females to replenish their energy stores (Gray et al., 2018; Keppner, Ayasse, & Steiger, 2018; Trumbo & Xhihani, 2015). In addition, starved females had fewer offspring alive at eclosion but only when breeding on a large carcass (see below for discussion of this interaction). Finally, resources acquired at the onset of breeding (i.e. carcass size) influenced multiple traits as females breeding on a large carcass produced more eggs, larger eggs, heavier broods, more larvae and heavier larvae than females that acquired a small carcass. These results are unsurprising given that the carcass acquired by the female is the only source of food for her offspring. In addition, females may adjust how many eggs they lay and how many offspring they rear to the amount of available resources (Bartlett, 1987; Bartlett & Ashworth, 1988; Müller et al., 1990). Taken together, our results demonstrate that variation in individual resource acquisition affects life‐history traits, but that limitation during different stages of the life cycle affects different traits and these differential effects make sense in the light of the biology of our study species.

We also found evidence for effects of the interaction between resource acquisition at different stages on life‐history traits. There was an interaction between resource acquisition during larval development and resource acquisition prior to breeding on egg size as females that acquired fewer resources during larval development and that were also starved prior to breeding laid larger eggs than females that received fewer resources during larval development but that were not starved prior to breeding. The proximate cause of this effect is unclear, but one potential explanation is that large females were able to lay large eggs regardless of their nutritional state, whereas small females may do so depending on how much they feed from the carcass. Thus, small females that were also starved may have produced larger eggs than small females that were not starved because starved females feed more from the carcass prior to commencing egg laying (Gray et al., 2018). In addition, starved females produced fewer offspring surviving to eclosion, but only when breeding on a large carcass. One potential explanation for this effect is that starved females spend less time suppressing microbial growth on large carcasses, which may elevate offspring mortality after dispersal (Gray et al., 2018). These results highlight that the effects of resource acquisition at a specific stage of the life cycle can be influenced by resource acquisition at other stages.

Our finding that there was a significant negative correlation between the number and size of offspring at dispersal only when females bred on small carcasses confirms that variation in resource acquisition at the start of breeding masks the trade‐off between offspring size and number. This finding is in agreement with previous work on this species (Smiseth et al., 2014) and suggests that females who acquire small carcasses face a trade‐off between the number and size of offspring that they produce, whereas females that acquire large carcasses do not. Carcass size likely had an effect on this trade‐off because the carcass acquired by the female represents the sole source of resources for reproduction, thereby determining how many resources are available for both offspring number and offspring growth. Our results contrast somewhat with Smiseth et al. (2014) who found that this relationship was weaker, but still negative, when females bred on a large carcass. This may reflect differences in experimental design between studies as we used larger ‘large’ carcasses (23–28 g; our study vs. 15–20 g; Smiseth et al., 2014). Thus, our results may reflect that females breeding on carcasses larger than 20 g maximized both the size and number of offspring without running out of resources. In support of this, we noticed that the entire carcass was always consumed when females bred on small carcasses, whereas this was often not the case when females bred on large carcasses (90 of 106 broods dispersed before consuming the entire carcass). This suggests that larvae reared on a large carcass are able to reach a threshold size and disperse before all available carrion is consumed. These results show that variation in resource acquisition during breeding masks the trade‐off between offspring size and number (van Noordwijk & de Jong, 1986).

We found no evidence that resource acquisition affected the trade‐off between number and size of eggs or between brood mass and lifespan (proxies for current and future reproduction, respectively). There are several potential explanations for why resource acquisition had no effect on these trade‐offs. Firstly, resource acquisition may not affect trade‐offs between life‐history traits if there is no trade‐off between them. For instance, the absence of a negative relationship between clutch size and egg size in our experiment suggests that females can invest more in egg size without reducing the number of eggs laid. This result is in keeping with previous studies, which also found no relationship between clutch size and egg size in this species (Monteith et al., 2012; Steiger, 2013). Our result, along with those of previous studies, suggests that the cost of producing eggs is low in *Nicrophorus* species, potentially because females acquire resources for egg laying by feeding from the carcass (Scott & Traniello, 1987; Trumbo, Borst, & Robinson, 1995).

Secondly, resource acquisition may have had no effect on life‐history trade‐offs because such trade‐offs involve multiple traits, some of which were not measured in our study. If so, the lack of evidence for a trade‐off between the proxy measures of current and future reproduction in our study (i.e. brood mass and lifespan, respectively) may reflect that allocating resources to current reproduction was associated with costs that were not measured or not detectable in a benign laboratory environment. For example, increased investment to current reproduction may induce reduced investment to immunity as reported in other species (e.g. Ilmonen, Taarna, & Hasselquist, 2000; Kraaijeveld, Limentani, & Godfray, 2001; Reaney & Knell, 2010; Simmons & Roberts, 2005), and reduced investment to immunity could in turn reduce survival and future reproduction in the wild where individuals are more likely to experience injury or infection. In *N. vespilloides*, there is evidence that exposure to infection shifts allocation towards current reproduction and away from survival (Cotter, Ward, & Kilner, 2011; Reavey, Silva, & Cotter, 2015), suggesting that there is a trade‐off between investing in current reproduction and immunity with subsequent effects on future reproduction.

Finally, resource acquisition may have had no effect on life‐history trade‐offs because of cryptic variation between individuals in some other aspect of their quality. The amount of resources an individual acquires is often treated as synonymous with an individual's quality (Bergeron, Baeta, Pelletier, Reale, & Garant, 2011; Wilson & Nussey, 2010). However, individuals that have acquired the same amount of resources may still differ in other respects, such as their ability to assimilate or utilize acquired resources. For instance, in *Daphnia pulicaria*, positive correlations between life‐history traits persist even when controlling for individual variation in resource acquisition because individuals differ in their ability to utilize resources (Olijnyk & Nelson, 2013). In sum, our results demonstrate that whereas individual variation in resource acquisition at different stages of the life cycle can have differential effects on life‐history traits, this is not necessarily associated with effects on trade‐offs between life‐history traits.

Our study adds to previous work suggesting that necrophagous, coprophagous and parasitoid insects are valuable study systems for investigating the effects of phenotypic variation in resource acquisition on life‐history decisions (e.g. Hunt, Simmons, & Kotiaho, 2002; Saeki & Crowley, 2013; Smiseth et al., 2014). This is because these systems allow for direct measurements and manipulations of variation in the amount of resources that parents acquire at the onset of reproduction (i.e. carcass size, dung pat size or host size). Our results suggest that in such systems, variation in the size of the resource has important consequences for life‐history traits and that it can mask trade‐offs between life‐history traits. In contrast, variation in resource acquisition prior to breeding has consequences for some life‐history traits but not for their associated trade‐offs. This appears to be the case regardless of whether such variation has fixed effects, as is the case in larval development, or temporary effects, as in the case of adult nutritional condition. As such, phenotypic variation in the resources acquired for breeding can have important consequences for life‐history traits and trade‐offs and may be important in determining how individuals cope with environmental instability. Although our results suggest that life‐history trade‐offs in *N. vespilloides* are only influenced by resource acquisition at the onset of breeding, we note that prior work shows that the ability of individuals to acquire such resources is determined by both their body size (Otronen, 1988) and their nutritional state (Hopwood et al., 2013). Thus, resource acquisition during different stages of the life cycle may have effects on life‐history trade‐offs by determining an individual's ability to access resources in the presence of environmental conditions, such as intraspecific competition.

## References

[jeb13388-bib-0001] Abràmoff, M. D. , Magalhães, P. J. & Ram, S. J. (2004). Image processing with ImageJ. Biophotonics International, 11, 36–42.

[jeb13388-bib-0002] Arce, A. , Johnston, P. , Smiseth, P. T. & Rozen, D. (2012). Mechanisms and fitness effects of antibacterial defences in a carrion beetle. Journal of Evolutionary Biology, 25, 930–937. 10.1111/j.1420-9101.2012.02486.x 22409257

[jeb13388-bib-0003] Barrett, E. L. B. , Hunt, J. , Moore, A. J. & Moore, P. J. (2009). Separate and combined effects of nutrition during juvenile and sexual development on female life‐history trajectories: The thrifty phenotype in a cockroach. Proceedings of the Royal Society B, 276, 3257–3264. 10.1098/rspb.2009.0725 19553255 PMC2817170

[jeb13388-bib-0004] Bartlett, J. (1987). Filial cannibalism in burying beetles. Behavioral Ecology and Sociobiology, 21, 179–183. 10.1007/BF00303208

[jeb13388-bib-0005] Bartlett, J. & Ashworth, C. M. (1988). Brood size and fitness in *Nicrophorus vespilloides* (Coleoptera: Silphidae). Behavioral Ecology and Sociobiology, 22, 429–434. 10.1007/BF00294981

[jeb13388-bib-0006] Benjamini, Y. & Hochberg, Y. (1995). Controlling the false discovery rate: A practical and powerful approach to multiple testing. Journal of the Royal Statistical Society. Series B (Methodological), 57, 289–300. 10.1111/j.2517-6161.1995.tb02031.x

[jeb13388-bib-0007] Bergeron, P. , Baeta, R. , Pelletier, F. , Reale, D. & Garant, D. (2011). Individual quality: Tautology or biological reality? Journal of Animal Ecology, 80, 361–364. 10.1111/j.1365-2656.2010.01770.x 21054382

[jeb13388-bib-0008] Berrigan, D. (1991). The allometry of egg size and number in insects. Oikos, 60, 313–321. 10.2307/3545073

[jeb13388-bib-0009] Botterill‐James, T. , Ford, L. , While, G. M. & Smiseth, P. T. (2017). Resource availability, but not polyandry, influences sibling conflict in a burying beetle *Nicrophorus vespilloides* . Behavioral Ecology, 28, 1093–1100. 10.1093/beheco/arx073

[jeb13388-bib-0010] Briga, M. , Koetsier, E. , Boonekamp, J. J. , Jimeno, B. & Verhulst, S. (2017). Food availability affects adult survival trajectories depending on early developmental conditions. Proceedings of the Royal Society B, 284, 20162287 10.1098/rspb.2016.2287 28053061 PMC5247499

[jeb13388-bib-0011] Brown, C. A. (2003). Offspring size‐number trade‐offs in scorpions: An empirical test of the van Noordwijk and de Jong model. Evolution, 57, 2184–2190. 10.1111/j.0014-3820.2003.tb00397.x 14575339

[jeb13388-bib-0012] Cotter, S. C. , Ward, R. J. & Kilner, R. M. (2011). Age‐specific reproductive investment in female burying beetles: Independent effects of state and risk of death. Functional Ecology, 25, 652–660. 10.1111/j.1365-2435.2010.01819.x

[jeb13388-bib-0013] Flatt, T. & Heyland, A. (2011). Mechanisms of life history evolution: The genetics and physiology of life history traits and trade‐offs. Oxford, UK: Oxford University Press 10.1093/acprof:oso/9780199568765.001.0001

[jeb13388-bib-0014] Ford, L. E. , Henderson, K. J. & Smiseth, P. T. (2018). Differential effects of offspring and maternal inbreeding on egg laying and offspring performance in the burying beetle *Nicrophorus vespilloides* . Journal of Evolutionary Biology, 31, 1047–1057. 10.1111/jeb.13285 29676514

[jeb13388-bib-0015] Ford, L. E. & Smiseth, P. T. (2016). Asynchronous hatching provides females with a means for increasing male care but incurs a cost by reducing offspring fitness. Journal of Evolutionary Biology, 29, 428–437. 10.1111/jeb.12797 26606605

[jeb13388-bib-0016] Gray, F. , Richardson, J. , Ratz, T. & Smiseth, P. T. (2018). No evidence for parent‐offspring competition in the burying beetle *Nicrophorus vespilloides* . Behavioral Ecology, 29, 1142–1149. 10.1093/beheco/ary091

[jeb13388-bib-0017] Hayward, A. D. , Rickard, I. J. & Lummaa, V. (2013). Influence of early‐life nutrition on mortality and reproductive success during a subsequent famine in a preindustrial population. Proceedings of the National Academy of Sciences of the United States of America, 110, 13886–13891. 10.1073/pnas.1301817110 23918366 PMC3752237

[jeb13388-bib-0018] Hopwood, P. E. , Moore, A. J. & Royle, N. J. (2013). Nutrition during sexual maturation affects competitive ability but not reproductive productivity in burying beetles. Functional Ecology, 27, 1350–1357. 10.1111/1365-2435.12137

[jeb13388-bib-0019] Hopwood, P. E. , Moore, A. J. & Royle, N. J. (2014). Effects of resource variation during early life and adult social environment on contest outcomes in burying beetles: A context‐dependent silver spoon strategy? Proceedings of the Royal Society of London, Series B: Biological Sciences, 281, 20133102 10.1098/rspb.2013.3102 24789890 PMC4024278

[jeb13388-bib-0020] Hunt, J. , Simmons, L. W. & Kotiaho, J. S. (2002). A cost of maternal care in the dung beetle Onthophagus taurus? Journal of Evolutionary Biology, 15, 57–64. 10.1046/j.1420-9101.2002.00374.x

[jeb13388-bib-0021] Ilmonen, P. , Taarna, T. & Hasselquist, D. (2000). Experimentally activated immune defence in female pied flycatchers results in reduced breeding success. Proceedings of the Royal Society B, 267, 665–670. 10.1098/rspb.2000.1053 10821610 PMC1690582

[jeb13388-bib-0022] Keppner, E. M. , Ayasse, M. & Steiger, S. (2018). Manipulation of parental nutritional condition reveals competition among family members. Journal of Evolutionary Biology, 31, 822–832. 10.1111/jeb.13266 29573021

[jeb13388-bib-0023] King, E. G. , Roff, D. A. & Fairbairn, D. J. (2011). Trade‐off acquisition and allocation in *Gryllus firmus*: A test of the Y model. Journal of Evolutionary Biology, 24, 256–264. 10.1111/j.1420-9101.2010.02160.x 21044204

[jeb13388-bib-0024] Kotrschal, A. , Szidat, S. & Taborsky, B. (2014). Developmental plasticity of growth and digestive efficiency in dependence of early‐life food availability. Functional Ecology, 28, 878–885. 10.1111/1365-2435.12230 25866430 PMC4384755

[jeb13388-bib-0025] Kraaijeveld, A. R. , Limentani, E. C. & Godfray, H. C. J. (2001). Basis of the trade–off between parasitoid resistance and larval competitive ability in *Drosophila melanogaster* . Proceedings of the Royal Society B, 268, 259–261. 11217895 10.1098/rspb.2000.1354 PMC1088600

[jeb13388-bib-0026] Lim, J. N. , Senior, A. M. & Nakagawa, S. (2014). Heterogeneity in individual quality and reproductive trade‐offs within species. Evolution, 68, 2306–2318. 24820133 10.1111/evo.12446

[jeb13388-bib-0027] Lindström, J. (1999). Early development and fitness in birds and mammals. Trends in Ecology & Evolution, 14, 343–348. 10.1016/S0169-5347(99)01639-0 10441307

[jeb13388-bib-0028] Lock, J. E. , Smiseth, P. T. & Moore, A. J. (2004). Selection, inheritance, and the evolution of parent‐offspring interactions. American Naturalist, 164, 13–24. 10.1086/421444 15266367

[jeb13388-bib-0029] Metcalfe, N. B. & Monaghan, P. (2001). Compensation for a bad start: Grow now, pay later?. Trends in Ecology & Evolution, 16, 254–260. 10.1016/S0169-5347(01)02124-3 11301155

[jeb13388-bib-0030] Monaghan, P. (2008). Early growth conditions, phenotypic development and environmental change. Philosophical Transactions of the Royal Society of London. Series B, Biological sciences, 363, 1635–1645. 10.1098/rstb.2007.0011 18048301 PMC2606729

[jeb13388-bib-0031] Monteith, K. M. , Andrews, C. & Smiseth, P. T. (2012). Post‐hatching parental care masks the effects of egg size on offspring fitness: A removal experiment on burying beetles. Journal of Evolutionary Biology, 25, 1815–1822. 10.1111/j.1420-9101.2012.02567.x 22775779

[jeb13388-bib-0032] Müller, J. K. , Eggert, A.‐K. & Furlkröger, E. (1990). Clutch size regulation in the burying beetle *Necrophorus vespilloides* Herbst (Coleoptera: Silphidae). Journal of Insect Behavior, 3, 265–270. 10.1007/BF01417917

[jeb13388-bib-0033] Nager, R. G. , Ruegger, C. & van Noordwijk, A. J. (1997). Nutrient or energy limitation on egg formation—a feeding experiment in great tits. Journal of Animal Ecology, 66, 495–507. 10.2307/5944

[jeb13388-bib-0034] Nagy, L. R. & Holmes, R. T. (2005). Food limits annual fecundity of a migratory songbird: An experimental study. Ecology, 86, 675–681. 10.1890/04-0155

[jeb13388-bib-0035] van Noordwijk, A. J. & de Jong, G. (1986). Acquisition and allocation of resources: Their influence on variation in life history tactics. American Naturalist, 128, 137–142. 10.1086/284547

[jeb13388-bib-0036] Olijnyk, A. M. & Nelson, W. A. (2013). Positive phenotypic correlations among life‐history traits remain in the absence of differential resource ingestion. Functional Ecology, 27, 165–172. 10.1111/1365-2435.12015

[jeb13388-bib-0037] Otronen, M. (1988). The effect of body size on the outcome of fights in burying beetles (Nicrophorus). Annales Zoologici Fennici, 25, 191–201.

[jeb13388-bib-0038] Pilakouta, N. , Halford, C. , Rácz, R. & Smiseth, P. T. (2016a). Effects of prior contest experience and contest outcome on female reproductive decisions and offspring fitness. American Naturalist, 188, 319–328. 10.1086/687392 27501089

[jeb13388-bib-0039] Pilakouta, N. , Richardson, J. & Smiseth, P. T. (2015). State‐dependent cooperation in burying beetles: Parents adjust their contribution towards care based on both their own and their partner's size. Journal of Evolutionary Biology, 28, 1965–1974. 10.1111/jeb.12712 26245748

[jeb13388-bib-0040] Pilakouta, N. , Richardson, J. & Smiseth, P. T. (2016b). If you eat, I eat: Resolution of sexual conflict over consumption from a shared resource. Animal Behavior, 111, 175–180. 10.1016/j.anbehav.2015.10.016

[jeb13388-bib-0041] Pilakouta, N. & Smiseth, P. T. (2016). Maternal effects alter the severity of inbreeding depression in the offspring. Proceedings of the Royal Society B, 283, 20161023 10.1098/rspb.2016.1023 27629026 PMC5031652

[jeb13388-bib-0042] R Core Team (2018). R: A language and environment for statistical computing. Vienna, Austria: R Foundation for Statistical Computing http://www.R-project.org/

[jeb13388-bib-0043] Reaney, L. T. & Knell, R. J. (2010). Immune activation but not male quality affects female current reproductive investment in a dung beetle. Behavioral Ecology, 21, 1367–1372. 10.1093/beheco/arq139

[jeb13388-bib-0044] Reavey, C. E. , Silva, F. W. & Cotter, S. C. (2015). Bacterial infection increases reproductive investment in burying beetles. Insects, 6, 926–942. 10.3390/insects6040926 26529021 PMC4693179

[jeb13388-bib-0045] Roff, D. (2002). Life history evolution. Sunderland, MA: Sinauer Associates.

[jeb13388-bib-0046] Saeki, Y. & Crowley, P. H. (2013). The size‐number trade‐off in clonal broods of a parasitic wasp: Responses to the amount and timing of resource availability. Functional Ecology, 27, 155–164. 10.1111/1365-2435.12014

[jeb13388-bib-0047] Safryn, S. A. & Scott, M. P. (2000). Sizing up the competition: Do burying beetles weigh or measure their opponents? Journal of Insect Behavior, 13, 291–297. 10.1023/A:1007700601095

[jeb13388-bib-0048] Sakai, S. & Harada, Y. (2001). Why do large mothers produce large offspring? Theory and a test. American Naturalist, 157, 348–359. 10.1086/319194 18707295

[jeb13388-bib-0049] Scott, M. P. (1994). The benefit of parental assistance in intra‐ and inter‐specific competition for the burying beetle, *Nicrophorus defodiens* . Ethology, Ecology & Evolution, 6, 537–543. 10.1080/08927014.1994.9522978

[jeb13388-bib-0050] Scott, M. P. (1998). The ecology and behavior of burying beetles. Annual Review of Entomology, 43, 595–618. 10.1146/annurev.ento.43.1.595 15012399

[jeb13388-bib-0051] Scott, M. P. & Traniello, J. F. A. (1987). Behavioral cues trigger ovarian development in the burying beetle, *Nicrophorus tomentosus* . Journal of Insect Physiology, 33, 693–696. 10.1016/0022-1910(87)90053-9

[jeb13388-bib-0052] Simmons, L. W. & Roberts, B. (2005). Bacterial immunity traded for sperm viability in male crickets. Science, 309, 2031 10.1126/science.1114500 16179472

[jeb13388-bib-0053] Smiseth, P. T. , Andrews, C. , Mattey, S. & Mooney, R. (2014). Phenotypic variation in resource acquisition influences trade‐off between number and mass of offspring in a burying beetle. Journal of Zoology, 293, 80–83. 10.1111/jzo.12115

[jeb13388-bib-0054] Smiseth, P. T. & Moore, A. J. (2002). Does resource availability affect offspring begging and parental provisioning in a partially begging species? Animal Behavior, 63, 577–585. 10.1006/anbe.2001.1944

[jeb13388-bib-0055] Smiseth, P. T. , Ward, R. S. J. & Moore, A. J. (2006). Asynchronous hatching in *Nicrophorus vespilloides*, an insect in which parents provide food for their offspring. Functional Ecology, 20, 151–156. 10.1111/j.1365-2435.2006.01072.x

[jeb13388-bib-0056] Smith, C. C. & Fretwell, S. D. (1974). The optimal balance between size and number of offspring. American Naturalist, 108, 499–506. 10.1086/282929

[jeb13388-bib-0057] Stearns, S. C. (1992). The evolution of life histories. Oxford, UK: Oxford University Press.

[jeb13388-bib-0058] Stearns, S. C. & Sage, R. D. (1980). Maladaptation in a marginal population of the mosquito fish, *Gambusia affinis* . Evolution, 34, 65–75. 10.1111/j.1558-5646.1980.tb04789.x 28563206

[jeb13388-bib-0059] Steiger, S. (2013). Bigger mothers are better mothers: Disentangling size‐related prenatal and postnatal maternal effects. Proceedings of the Royal Society B, 280, 20131225 10.1098/rspb.2013.1225 23843390 PMC3730594

[jeb13388-bib-0060] Steiger, S. , Richter, K. , Müller, J. K. & Eggert, A. K. (2007). Maternal nutritional condition and genetic differentiation affect brood size and offspring body size in Nicrophorus. Zoology, 110, 360–368. 10.1016/j.zool.2007.06.001 17702555

[jeb13388-bib-0061] Taborsky, B. (2006). The influence of juvenile and adult environments on life‐history trajectories. Proceedings of the Royal Society B, 273, 741–750. 10.1098/rspb.2005.3347 16608695 PMC1560075

[jeb13388-bib-0062] Trumbo, S. T. , Borst, D. W. & Robinson, G. E. (1995). Rapid elevation of juvenile hormone titer during behavioral assessment of the breeding resource by the burying beetle, *Nicrophorus orbicollis* . Journal of Insect Physiology, 41, 535–543. 10.1016/0022-1910(94)00127-3

[jeb13388-bib-0063] Trumbo, S. T. & Xhihani, E. (2015). Influences of parental care and food deprivation on regulation of body mass in a burying beetle. Ethology, 121, 985–993. 10.1111/eth.12413

[jeb13388-bib-0064] Wilson, A. J. & Nussey, D. H. (2010). What is individual quality? An evolutionary perspective Trends in Ecology & Evolution, 25, 207–214. 10.1016/j.tree.2009.10.002 19897275

[jeb13388-bib-0065] Wong, J. W. & Kölliker, M. (2014). Effects of food restriction across stages of juvenile and early adult development on body weight, survival and adult life history. Journal of Evolutionary Biology, 27, 2420–2430. 10.1111/jeb.12484 25263828

[jeb13388-bib-0066] Yanagi, S.‐I. & Tuda, M. (2012). Female size constrains egg size via the influence of reproductive organ size and resource storage in the seed beetle *Callosobruchus chinensis* . Journal of Insect Physiology, 58, 1432–1437. 10.1016/j.jinsphys.2012.08.007 23000737

[jeb13388-bib-0067] Zajitschek, F. , Hunt, J. , Jennions, M. D. , Hall, M. D. & Brooks, R. C. (2009). Effects of juvenile and adult diet on ageing and reproductive effort of male and female black field crickets, *Teleogryllus commodus* . Functional Ecology, 23, 602–611. 10.1111/j.1365-2435.2008.01520.x

[jeb13388-bib-0068] Zanette, L. , Clinchy, M. & Smith, J. N. M. (2006). Food and predators affect egg production in song sparrows. Ecology, 87, 2459–2467. 10.1890/0012-9658(2006)87[2459:FAPAEP]2.0.CO;2 17089655

